# Supplementary material for: A Likelihood Approach for Real-Time Calibration of Stochastic Compartmental Epidemic Models
Source: PLoS Comput Biol. 2017 Jan 17;13(1):e1005257. doi: 10.1371/journal.pcbi.1005257 (PMC5240920; doi:10.1371/journal.pcbi.1005257)
Supplement: S1 File — (TAR.GZ) [file pcbi.1005257.s014.tar.gz › HSPH_Online-SI-Revision/Additions/IPoi10/n100-extreme-SEITR/n100-extreme-SEITR_BENCH2_summary.pdf]

peak -8	3w spec	3w cum	5w spec	5w cum	end	3w spec LOG	3w cum LOG	5w spec LOG	5w cum LOG	end LOG
mean	88.4	88.4	86.5	87.5	51.5	1.9	1.7	2.5	2.2	1.9
median	98.	97.1	99.5	99.2	71.	2.4	2.1	3.4	2.9	2.2
quantile .1	63.5	63.9	49.3	58.9	5.2	0.4	0.4	0.4	0.4	0.
quantile .9	99.4	98.9	99.9	99.7	85.7	2.9	2.6	3.9	0.4	4.2
8	11	6	8	2						

peak -4	3w spec	3w cum	5w spec	5w cum	end	3w spec LOG	3w cum LOG	5w spec LOG	5w cum LOG	end LOG
mean	46.1	49.6	31.5	38.2	8.6	0.4	0.5	0.2	0.3	0.
median	38.2	40.6	26.8	31.7	7.	0.3	0.3	0.2	0.2	0.
quantile .1	16.1	24.	16.4	12.	3.8	0.1	0.1	0.1	0.1	0.
quantile .9	82.3	82.3	47.4	70.5	14.1	1.	1.	0.4	0.1	0.1
10	25	9	9	12						

peak	3w spec	3w cum	5w spec	5w cum	end	3w spec LOG	3w cum LOG	5w spec LOG	5w cum LOG	end LOG
mean	41.8	29.4	68.3	32.2	35.9	0.2	0.2	0.3	0.2	0.2
median	38.4	26.7	52.	28.6	33.	0.2	0.1	0.2	0.1	0.1
quantile .1	27.1	15.9	36.8	20.7	24.2	0.1	0.1	0.2	0.1	0.1
quantile .9	60.3	43.8	132.3	44.1	48.4	0.3	0.3	0.4	0.1	0.3
42	38	33	42	43						

peak +4	3w spec	3w cum	5w spec	5w cum	end	3w spec LOG	3w cum LOG	5w spec LOG	5w cum LOG	end LOG
mean	138.5	99.3	229.4	109.	119.5	0.3	0.3	0.4	0.3	0.3
median	83.5	61.7	108.	68.1	87.8	0.3	0.2	0.3	0.2	0.3
quantile .1	43.8	40.2	45.6	42.1	42.3	0.2	0.2	0.2	0.2	0.2
quantile .9	257.9	162.9	478.8	182.9	203.	0.5	0.5	0.7	0.2	0.5
25	27	21	28	25						

peak +8	3w spec	3w cum	5w spec	5w cum	end	3w spec LOG	3w cum LOG	5w spec LOG	5w cum LOG	end LOG
mean	661.3	227.6	2268.	247.9	268.5	0.5	0.4	0.6	0.4	0.4
median	156.6	125.	179.1	132.	141.8	0.4	0.3	0.4	0.3	0.4
quantile .1	46.3	45.1	48.1	45.4	46.3	0.2	0.2	0.2	0.2	0.2
quantile .9	783.9	452.7	1400.	522.7	587.8	0.8	0.7	1.1	0.2	0.7
18	22	19	18	17	{}	{}	{}	{}	{}	{}
